# Mammary fat globules as a source of mRNA to model alterations in the expression of some milk component genes during lactation in bovines

**DOI:** 10.1186/s12917-024-04130-8

**Published:** 2024-07-03

**Authors:** Samar M. Shaban, Rania A. Hassan, Abeer A. I. Hassanin, Ahmed Fathy, Adel A. S. El Nabtiti

**Affiliations:** 1 Department of Animal Wealth Development (Animal Production Division), Faculty of Veterinary Medicine, Suez Canal University, Ismailia, 41522 Egypt; 2 Department of Animal Wealth Development (Genetics and Genetic Engineering Branch), Faculty of Veterinary Medicine, Suez Canal University, Ismailia, 41522 Egypt; 3 Department of Animal Wealth Development (Biostatistics Division), Faculty of Veterinary Medicine, Suez Canal University, Ismailia, 41522 Egypt

**Keywords:** Gene expression, Milk composition, Egyptian buffalo, Holstein Friesian

## Abstract

**Background:**

The milk's nutritional value is determined by its constituents, including fat, protein, carbohydrates, and minerals. The mammary gland's ability to produce milk is controlled by a complex network of genes.

Thereby, the fat, protein, and lactose synthesis must be boost in milk to increase milk production efficiency. This can be accomplished by fusing genetic advancements with proper management practices. Therefore, this study aimed to investigate the association between the Lipoprotein lipase (LPL), kappa casein CSN3, and Glucose transporter 1 (GLUT1) genes expression levels and such milk components as fat, protein, and lactose in different dairy breeds during different stages of lactation.

**Methods:**

To achieve such a purpose, 94 milk samples were collected (72 samples from 36 multiparous black-white and red-white Holstein–Friesian (HF) cows and 22 milk samples from 11 Egyptian buffaloes) during the early and peak lactation stages. The milk samples were utilized for milk analysis and genes expressions analyses using non- invasive approach in obtaining milk fat globules (MFGs) as a source of Ribonucleic acid (RNA).

**Results:**

LPL and CSN3 genes expressions levels were found to be significantly higher in Egyptian buffalo than Holstein–Friesian (HF) cows as well as fat and protein percentages. On the other hand, GLUT1 gene expression level was shown to be significantly higher during peak lactation than early lactation. Moreover, lactose % showed a significant difference in peak lactation phase compared to early lactation phase. Also, fat and protein percentages were significantly higher in early lactation period than peak lactation period but lactose% showed the opposite pattern of Egyptian buffalo.

**Conclusion:**

Total RNA can be successfully obtained from MFGs. The results suggest that these genes play a role in glucose absorption and lactose synthesis in bovine mammary epithelial cells during lactation. Also, these results provide light on the differential expression of these genes among distinct Holstein–Friesian cow breeds and Egyptian buffalo subspecies throughout various lactation phases.

## Background

Many stakeholders in the milk industry, including farmers, processors, and consumers, place a premium on knowing exactly what's in their product due to health concerns and because the combination of milk ingredients affects the milk industry's worldwide pricing strategy [1]. In general, milk is comprised of 87.7% water, 3.4% fat, 3.3% protein, 4.9% lactose, and 0.7% mineral matter [2].

Among all the components, lipids show the highest variation among breeds [3]. Milk fat was mostly composed of triglycerides, with only a small amount of other lipids [4]. According to [5], milk fat has dual sources; fatty acids are either generated de novo from short, medium-chain fatty acids in the mammary glands or obtained from dietary long-chain fatty acids (LCFA). Lipoprotein lipase (LPL) is created in the mammary gland's epithelial cells and impacts fatty acid release in the mammary gland, since LPL activity becomes greater in the lactating mammary gland [6, 7]. LPL enzyme releases these LCFA from circulating triglycerides in chylomicrons or VLDL, then imports them from the plasma [8]. Lactose, a disaccharide composed of glucose and galactose, is the primary carbohydrate in milk [9]. Milk lactose has numerous uses, including preserving milk osmolarity with the help of dissolved inorganic ions (Na + , K + , Cl) [10]. Milk protein is divided in to both casein and whey protein, the two types are dissimilar in their solubility at pH 4.6 and 20 °C [11–13]. Milk protein composition varies widely between species [14], but there is a strong correlation between the amount of protein in maternal milk and the rate at which a species' newborns develop [15].

The composition, characteristics, and structures of milk components can change greatly based on a wide range of conditions such as breed, species, parity, and lactation order [16, 17]. Therefore, adopting suitable breeding systems that improve dairy animal output and alter milk quality, it is crucial to pay close attention to the relative impact of both genetic and environmental influences. Increases in milk fat, protein, and lactose content can be achieved through a combination of genetics and effective management that targets the nutritional content of the important molecules utilized by the mammary gland to produce milk [18].

Gene expression plays an important part in the long-term cellular metabolism processes of many dairy animals, including the Holstein cow and the buffalo [19]. Several studies have demonstrated the importance of gene expression analyses for the genes that relate to milk components such as Lipoprotein lipase (LPL), Kappa Casein (CSN3), and Glucose transporter 1 gene (GLUT1) at different lactation stages [20, 21].

These genes play important role in milk constituents synthesis where, LPL encoded by LPL gene, (protein coding gene) where, Triacylglycerol hydrolysis and free fatty acids uptake from the plasma are crucial processes that are facilitated by LPL [22]. Moreever, All the major milk proteins are synthesized in mammary gland and encoded by milk protein genes, CSN3 gene is one of them [23], that encodes kappa casein protein. it is an important milk protein for the structure, formation and stability of casein micelles with regard to cheese processing and technical features of milk [24–26]. For GLUT1, GLUT1 is the principal glucose transporter in the dairy cattles’ mammary epithelial cells that accountable for basal glucose uptake where the main precursor of lactose is glucose [27, 28].

According to [29] the use of gene expression analysis in livestock species is growing because it advances our knowledge of intricate biological processes like lactation physiology. A actual picture of the molecular processes associated with milk synthesis during lactation can be obtained by examining the expression profiles of genes linked to milk synthesis through RNA isolation from milk fat globules(MFGs) rather than mammary tissue, as has been highlighted by studies conducted in various species [30]**.** Breeders' main focus in their investigations into the factors influencing the differential production potential of various breeds continued to be genetic variability to design suitable breeding program improving milk production.

Traditionally, mammary biopsies have been used to study gene expression in the mammary gland of dairy animals. These biopsies are invasive, painful, and expensive, and they also disrupt the normal lactation process [31–33]. These biopsies are avoided through MFGs where, Mammary Epithelial Cells (MECs) are responsible for the synthesis of milk fat during lactation [34]. Milk fat globules (MFGs) are secreted from MECs into milk via an apocrine mechanism that involves a crescent of MEC cytoplasm encased in plasma membrane [35, 36]. The MFGs become entrapped with MEC intracellular components [37]. Therefore, prior investigations have shown that RNA extracted from MFGs is indicative of RNA extracted from MECs during milk production [32, 38, 39]. Therefore, MFGs in milk can be used as reliable and accessible sources for investigating lactation-related gene expression.

Therefore, the purpose of this research was to examine the relationship between the expression of LPL, CSN3, and GLUT1genes and the levels of fat, protein, and lactose in MFGs respectively during different stages of lacta tion in two different dairy animal species / breeds (Egyptian Buffalo and Holstein–Friesian).

## Results

The MFGs were used as source of total RNA from Egyptian Buffaloes and two breeds of HF cattle at different lactation phases (Early and Peak) using GeneZol™ CT RNA isolation reagent (puregene, Genetex Biotech Asia Pvt.Ltd.) method exhibited high quality RNA where, the A260/280 ratio was in the range of 1·78 to 1.9 with Adequate concentration in the range of 287.7:310.9ng/µl.

The investigation of the alterations in the expression of some milk component genes (LPL, CSN3, and GLUT1) during lactation in bovines was done through the effect of different dairy breeds (Egyptian buffaloes, Black/white HF cow, and Red/white HF cow) and lactation stage (early, and peak lactation phases) as fixed factors on gene expression (LPL, CSN3, and GLUT1) and milk composition (fat%, protein %, and Lactose%).

### Effect of dairy breeds and stage of lactation on gene expression levels

**Fig. 1:**
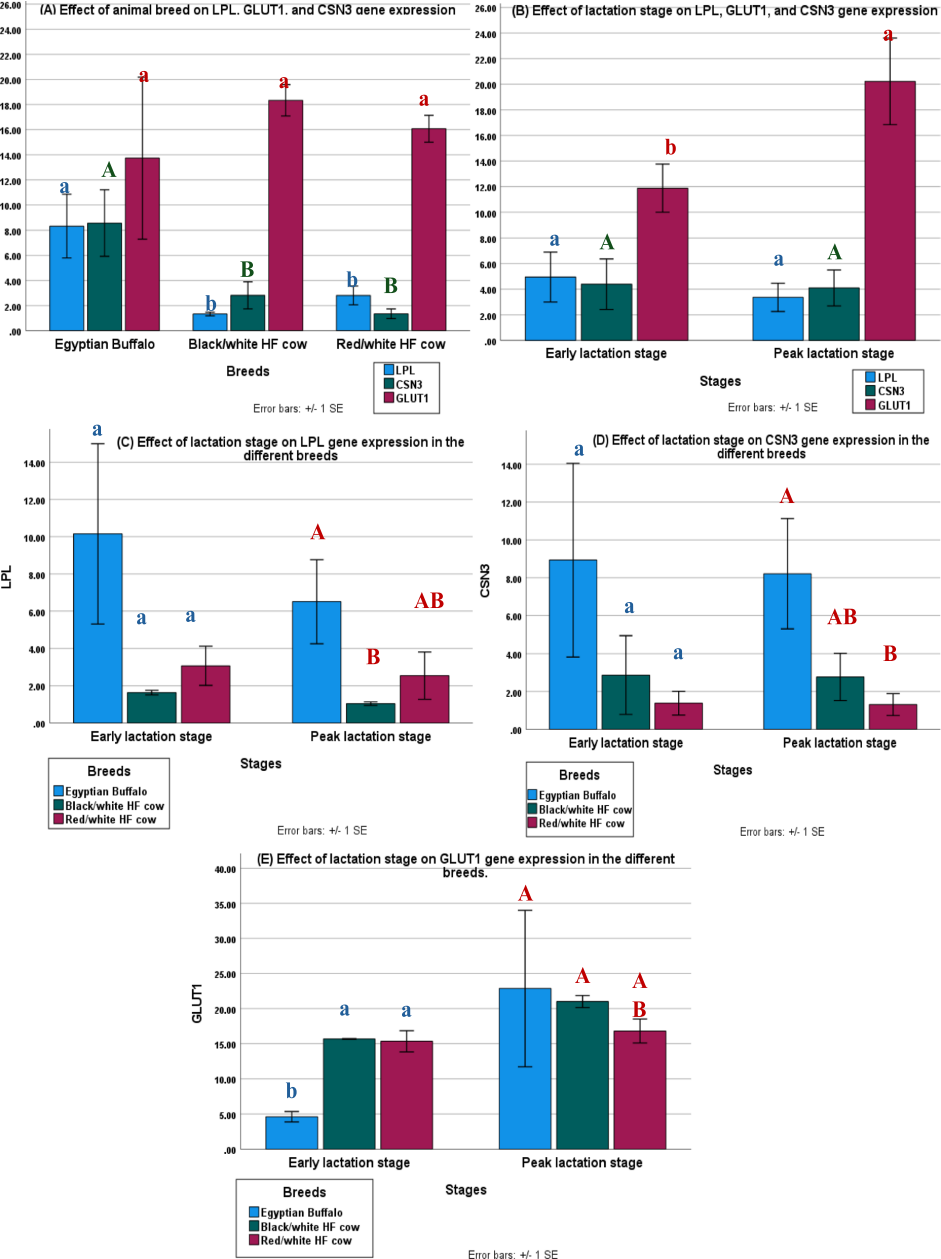
Effect of dairy breeds and stage of lactation on gene expression levels: **A** Effect of animal breed on LPL, G1, and CSN3 gene expression. **B** Effect of lactation stage on LPL, GLUT1, and CSN3 gene expression. **C** Effect of lactation stage on LPL gene expression in the different breeds. **D** Effect of lactation stage on CSN3 gene expression in the different breeds. **E** Effect of lactation stage on GLUT1 gene expression in the different breeds. means of different letters are statistically significant at *p* < 0.01

The results presented that the expression levels of LPL (8.33 ± 2.52) and CSN3 (8.58 ± 2.64) were significantly higher in Egyptian buffalo than in other dairy breeds under the study (Fig. 1A). However, the expression level of the GLUT1 gene did not differ significantly between various dairy breeds under the study (Fig. 1A).The expression profiles of LPL and CSN3 genes did not revealed significant difference btween two lactation phases. Although, their expression were higher in early lactation phase than peak lactation phase (Fig. 1B). However, GLUT1 gene expression level appears to be significantly higher during peak lactation phase (20.23 ± 3.38) than during early lactation phase (11.88 ± 1.88) (Fig. 1B).

CSN3 gene expression did not vary significantly between dairy breeds in early lactation stage (Fig. 1D). also, the expression level of LPL gene was higher in early lactation phase than peak lactation phase (Fig. 1C). As shown in Fig. 1E, GLUT1 gene expression was higher in the peak lactation stage (22.87 ± 11.14) compared to early lactation stage (4.62 ± 0.74) of Egyptian buffalo. But, it did not show significant differences between Black/white and Red/white HF cows in both lacation phases. although, its expression level was higher during peak lactation stage than early lactation phase in two HF cows.

### Effect of dairy breeds and stage of lactation on milk components

**Fig. 2:**
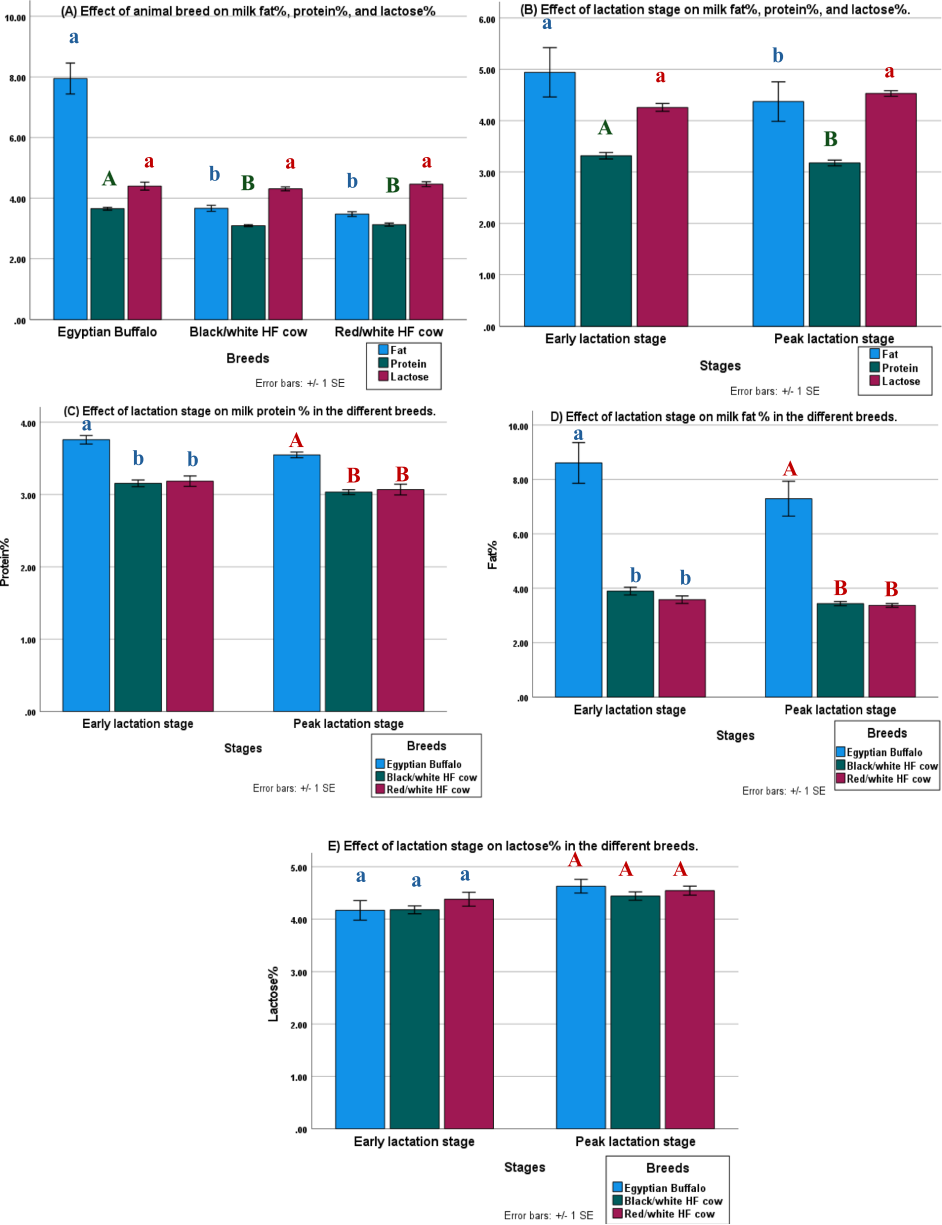
Effect of dairy breeds and stage of lactation on some milk components: **A** Effect of animal breed on milk fat%, protein%, and lactose%. **B** Effect of lactation stage on milk fat%, protein%, and lactose%. **C** Effect of lactation stage on milk protein % in the different breeds. **D** Effect of lactation stage on milk fat% in the different breeds. **E** Effect of lactation stage on lactose% in the different breeds. means of different letters are statistically significant at *p* < 0.01

As shown in Fig. 2A, the fat and protein contents of Egyptian buffalo milk were significantly higher than that of other breeds involved in the resaerch. However, lactose content analysis did not show significant differences in all animals included in the study. The percentages of milk fat and milk protein were found to be significantly higher during early lactation period compared to peak lactation period (Fig. 2B). Furthermore, as can be shown in Fig. 2B, lactose% exhibited an increase during the peak lactation period compared to the early lactation period. Milk analysis for protein and fat percentages showed significant superiority for Egyptian buffalo over the two breeds of Black/white and Red/white HF cow in both the early lactation stage and the peak lactation stage (Figs. 2C and D). In the peak lactation period, the percentage of lactose produced by various dairy breeds was much higher than in the early lactation stage (Fig. 2E).

### Prediction of milk components based on levels of gene expression using multivariate regression model

The tested assumption of the multivariate regression model, which was developed to test the relationship between gene expression and the level of fat%, protein%, and lactose%, as milk components, were nearly fully met. Kolmogorov–Smirnov and Shapiro test examined the normality assumption for residuals, the test statistic for Kolmogorov–Smirnov ranged between 0.103 and 0.144 with *p-value* = 0.200, while that of Shapiro ranged between 0.937 and 0.957 with *p-value* > 0.05, and these results support normality assumption. The multivariate extremes checked by Mahalanobis distance (Minimum Mahalanobis = 0.159, Maximum Mahalanobis = 12.751, Chi-square = 23.685 and *p-value* > 0.05), showed absence of outliers. Scatter plots and ANOVA (*P* < 0.05) revealed a linear relationship between the dependent variables and each of the predictor variables. The multicollinearity diagnostic statistics results showed that the tolerance ranged between 0.570, and 0.795 which is not less than 0.2 and not cause any multicollinearity problem, the variance inflation factor ranged between 1.258, and 1.755, and these values lies between 1 and 5 indicate that the correlation between predictor variables is moderate.

Multiple linear regression models for forecasting of milk composition parameters based on information in Table 1, as follow:

$$\begin{array}{c}Fat\%=0.024+0.461G_{LPL}+0.313G_{CSN3}+0.126G_{GLUT1}\\\Pr otein\%=2.972+0.046G_{LPL}+0.023G_{CSN3}+0.007G_{GLUT1}\\Lactose\%=4.258+0.008G_{LPL}+0.012G_{CSN3}+0.008G_{GLUT1}\end{array}$$

**Table 1:** Multiple linear regression co-efficient for prediction of milk compositions

Predictors	Biochemical blood parameter**s**	Unstandardized coefficients		Standardized coefficints	t	sig
		β	S.E	Beta		
**Constant**	**Fat%**	0.024	0.787		0.030	0.976
	**Protein%**	2.972	0.163		18.265	< 0.001
	**Lactose**	4.258	0.239		17.815	< 0.001
LPL	**Fat%**	0.461	0.056	0.915	8.228	< 0.001
	**Protein%**	0.046	0.012	0.782	3.945	0.001
	**Lactose**	0.008	0.017	0.141	0.454	0.657
CSN3	**Fat%**	0.313	0.050	0.644	6.217	< 0.001
	**Protein%**	0.023	0.010	0.404	2.187	0.046
	**Lactose**	0.012	0.015	0.229	0.789	0.443
GLUT1	**Fat%**	0.126	0.032	0.484	3.956	0.001
	**Protein%**	0.007	0.007	0.243	1.114	0.284
	**Lactose**	0.008	0.010	0.289	0.843	0.414

The coefficient of determination determines the goodness of fit. Parameter values for goodness of fit are mentioned in Table 2. R^2^value was the largest between the predictor variables and fat% as a milk component parameter by 0.881, it means that the proportion of variation in fat% caused by the predictors was 88.1%, on the other hand the weakest correlation was observed between the predictor parameters and lactose% with 0.25 and the percent of variation in lactose caused by gene expressions was 6.2% with R^2^ = 0.062.

**Table 2:** Multiple linear regression predictive model summary for milk compositions

Model	R	R Square	Adjusted R Square	Std. Error of the Estimate	Change Statistics				
					R Square Change	F Change	df1	df2	Sig. F Change
**Fat%**	0.938	0.881	0.855	0.897	0.881	34.387	3	14	< 0.001
**Protein%**	0.788	0.620	0.539	0.185	0.620	7.621	3	14	0.003
**Lactose**	0.250	0.062	-0.139	0.272	0.062	0.310	3	14	0.818

## Discussion

The gene expression patterns of MFG-derived RNA are more representative than MEC-derived RNA because MFGs can be obtained at any time throughout lactation and RNA extraction from MFGs is less complicated [32, 40]. MECs produce milk lipid [34] and secrete it as milk lipid globules (MFGs) to myoepithelial cells, fibroblasts, blood vessels, adipocytes, and immune cells via an apocrine mechanism. Therefore, RNA and other intracellular components of MECs are ingested by MFGs [41]. In addition to MECs, several cell types can be found in mammary tissue [33, 42] which means that RNA isolated from mammary tissue cannot be used to reliably identify gene expression in MECs. Besides that, Mammary biopsy is an invasive procedure that can interrupt lactation.

According to our data, LPL gene expression is highest in Egyptian Buffalo compared to the two HF breeds specially at early lactation stage compared to peak stage. These findings were consistent with those of M Janmeda, V Kharadi, G Pandya, B Brahmkshtri, U Ramani and K Tyagi [43], who found that, in Surti buffalo, LPL expression was highest on 15th day after calving, then decreased till day 60. Non-esterified free Fatty acids (NEFA) and lipoproteins supply long chain fatty acids (LCFA) to mammary cells, this may explain why the LPL gene is expressed at a higher level during early lactation. By attaching chylomicrons or VLDL to the mammary endothelium, lipoprotein lipase can digest TAG center of the lipoprotein to release FA that can be used in milk fat synthesis. Additionally, TAG absorption efficiency from lipoprotein in the mammary gland is enhanced at the onset of lactation [7, 19].

In contrary, S Wickramasinghe, G Rincon, A Islas-Trejo and JF Medrano [44] reported that LPL gene expression was highest in Holstein cows during peak lactation stage [DIM = 90 days], and quite high during the transition [DIM = 15 days] stage of lactation. As well as, E Sandri, K Harvatine and D Oliveira [20] found that LPL gene expression in Holstein cattle was highest during the peak [DIM = 60 days] stage of lactation and subsequently dropped during the late stage of lactation. Jaffarabadi buffalo, on the other hand, showed no variation in LPL expression between days 15 and 60 of lactation (M Janmeda, V Kharadi, G Pandya, B Brahmkshtri, U Ramani and K Tyagi [43]. Similarities between the lactation curve and LPL expression suggest a potential role for this gene in sustaining milk synthesis.

The kappa casein protein encoded by the CSN3 gene is a kind of milk casein (casein). This study found that CSN3 expression was highest in the early lactation phase and then decreased throughout the latter stages of lactation, therefore milk protein concentration was higher in the first part of lactation compared to later stages. Enhanced expression of this gene is associated with milk protein production as casein protein accounts for up to 80% of the protein in milk, whereas whey protein accounts for up to 20% [18]. During lactation, the mammary glands alveolar epithelial cells synthesize milk's protein, and certain immunoglobulins are directly transported from the blood into milk without changes [23].

Our results were in agreement with those of Arora et al. [45], who found that CSN3 expression in Murrah buffalo was highest in the early lactation and gradually decreased thereafter (which include peak lactation). Furthermore, many studies have shown that the maximum concentration of proteins is found in colostrum and in the early stages of lactation [42, 46]. Furthermore, Wickramasinghe et al. [44] demonstrated that CSN3 gene expression was highest during transition lactation and decreased significantly later in lactation (involving peak lactation point). Our results also were similar to the observations of Contreras et al. [47], who reported that the CSN3 gene showed the highest expression on ( 8th and 15th days) post-calving (the first two weeks of lactation).

The present study found that Black/white, Red/white HF, and Egyptian buffaloes expressed their GLUT1 genes in a similar manner with the recorded milk data, in which, it increases with the increasing of milk production and reaches its peak between the 50th and 60th days after calving. These findings corroborated with results of Zhao and Keating [48] who investigated that the pattern of GLUT1 gene expression generally followed the lactation curve. The expression of GLUT1 increases rapidly (DIM = 10–20 days) during the first three weeks of lactation. At peak lactation, GLUT1 mRNA levels remained high. In order to meet the demands of milk synthesis, the mammary glands glucose requirement increases dramatically throughout the early and later stages of lactation, a phenomenon that may be linked to increased GLUT1 mRNA expression in MEC. This coincides with Dänicke et al. [49] findings, that demonstrated GLUT1 expression in the mammary glands at peak and late lactation in lactating cows, but barely detectable levels in non-lactating cows.

According to a study conducted by Yadavet al. [30], the expression of GLUT1 in Riverine buffalo (Bubalus bubalis) correlated positively with milk production at both the early (1.01 mg/dl) and late (4.68 mg/dl) phases of lactation. However, Mann et al. [50] reported the same level of GLUT1 expression in both stages of lactation (early, peak). On the other hand, Pradeep et al. [51] showed that GLUT1 gene expression was highest during the cows' peak lactation stage and lowest during the cows' early lactation stage. Compared to buffalo, GLUT1 gene expression was found to be higher in cows, as reported by both Yadav et al. [30] and Pradeep et al. [51]. Since the quantity of milk produced is directly proportional to the amount of glucose absorbed by the alveolar epithelial cells of the mammary gland Pradeep et al. [51], and since GLUT1 is the predominant glucose transporter that is responsible for glucose transmission in the mammary gland, the GLUT1 expression would be correlated with milk production throughout the two stages of lactation.

Milk quantity is indicative of the amount of glucose absorption by the mammary epithelial cells (MEC), which is directly proportional to the number of glucose transporter molecules present on the surface of MEC, indicating an indirect relationship between milk quantity and glucose transport. The lactose synthesis occurring in the Golgi apparatus controls the fate of glucose metabolism in MEC [52, 53]. In the mammary gland, glucose absorption is dominated by the GLUT transporter group. As lactation begins, glucose absorption in the mammary gland increases rapidly, and the expression of these transporters has been connected to milk production. Lactose production affects milk quantity and composition as well [54]. Previously, investigations on cows had suggested a linear relationship between glucose intake and milk production [55].

The present findings regarding milk components, the analyses reported a higher percentage of fat and protein in the early stage than in the peak period. Milk fat and milk protein percentages dropped throughout the first 8wks post-calving [47, 56]. The lowest percentages of milk fat and milk protein were produced by Danish Holstein–Friesian Cows 40 and 60 days after calving [56]. Similar to what was shown by Bhat et al. [57], we found that the lactose % in milk remained stable for the first 20 weeks of lactation but seemed to increase on day 57.

The present analyses results for lactose, were agreed with, results noted by Sigl et al., [56], that lactose percentage remained constant throughout the initial 20 weeks of lactation, and seemed to be elevated by 57th day. Nearly similar observation was in Israeli Holstein–Friesian cows on DIM = 66, in which, lactose reached maximum level [58].

## Conclusion

This study sought to determine the association between the expression of the LPL, CSN3, and GLUT1 genes and the amounts of fat, protein, and lactose, respectively, utilizing MFGs as sources of RNA in Egyptian buffalo and two breeds of HF cows during various lactation phases. The obtained results revealed that the expression profiles of the LPL and CSN3 genes were highly significant compared to two breeds of HF cows, as the results of fat% and protein% in Egyptian buffalo. Moreover, GLUT1 gene expression and lactose% manifested highly significant differences in peak lactation phase compared to early lactation phase. These results suggest that the LPL and GLUT1 genes have a functional role in milk fat and lactose synthesis, respectively, in bovine mammary epithelial cells during lactation. As well as, it aided in the explanation of the pattern of LPL, CSN3, and GLUT1 gene expression at distinct lactation phases in Egyptian buffalo and HF cattle. Therby, understanding the transcription of the genes involved in the milk synthesis process, it may be possible to improve the efficiency of milk component synthesis. Also, to produce pure, healthy, and nutritious milk and dairy products, more attention should be focused on milk components as a selection trait in breeding strategies. Producing high-quality milk and milk products would result in higher profits for their company (the producer). These findings need to be confirmed and verified by additional research.

## Methods

This research was performed at Animal Wealth Development Department, Veterinary Medicine Faculty, Suez Canal University, Ismailia, Egypt.

### Experimental design

Egyptian buffaloes (11 buffaloes) and two breeds of HF cows (19 Black/white HF breed and 17 Red/white HF breed) were utilized for milk sample collection at two various lactation phases; early phase DIM = 10:20 days, peak phase DIM = 45:60 days. The samples had been intended for milk analysis and total RNA extraction from MFGs, which was followed by cDNA synthesis and quantitative RT-PCR using specific primers to investigate the gene expression of the LPL gene, which codes for the LPL enzyme responsible for triacylglycerol hydrolysis and free fatty acid uptake by mammary epithelial cells for milk fat synthesis, the CSN3 gene, which codes for the important milk protein kappa-casein, and, lastly, the GLUT1 gene, which codes for glucose transporter 1 that responsible for glucose transportation through the MECs for lactose synthesis.

### Animals involved in the Study

36 healthy multiparous cows (19 Black/white HF cows and 17 Red/white HF cows) in their third lactation season from a local government farm in Egypt were used in this study. They were all raised under the same management and feeding system, all cows were fed a total mixed ration (TMR) with variable roughage-to-concentrate ratios based on their productive phases. The concentrate portion consisted of soybean meal, yellow maize, sunflower meal, distilled dried grains, and wheat bran, while the roughage portion for buffalo consisted of silage and clover in the winter and sorghum, wheat straw, and rice straw in the summer. Furthermore, Eleven Egyptian buffaloes were recruited from the veterinary medicine faculty farm at Suez Canal University in Ismailia, Egypt. The animals were fed a tsotal mixed ration (TMR) with variable roughage-to-concentrate ratios dependent on their productive periods, and they had free access to water. The cows in the study are milked three times daily by an automatic milking system, while the buffaloes are milked manually twice daily.

### Milk samples

Early in the morning (7–8 a.m.), during the milking process, 72 milk samples were obtained from both HF cows and 11 Egyptian buffaloes at two periods of lactation: early stage (10–20 days post calving) and peak stage (50-60 days post calving). The udder was washed, dried, and disinfected, and the milking process was completed. A total of 115 ml of milk from the whole milk of each cow and buffalo was collected under hygienic conditions by graduated container, with 100 ml destined for RNA isolation and 15 ml destined for milk analysis. 100 ml-sample was divided evenly between two 50 ml-sterile falcon tubes (for centrifugation and milk fat pooling) and rushed to the lab in an ice box for analysis and isolation within 4 h.

### Milk analysis

The LACTOSCAN system (ULTRASONIC MILK ANALYZER, 30 Second Lactoscan SLP Milk Analyzer, Ltd./Pvt. Ltd, India) was used to estimate the percentages of milk fat, milk protein, and lactose according to the manufacturer's procedure.

### Sample preparation, RNA extraction, and reverse transcription

The milk samples were centrifuged at 4 °C at 2000 rpm for 20 min for fat phase separation. GeneZolTM CT RNA isolation reagent (puregene, Genetex Biotech Asia Pvt.Ltd.) was used to isolate RNA from milk fat samples in the following steps [59]. 1.5 ml of genezol reagent /0.5 g of milk fat [60] was added, followed by vortexing for homogenization. The homogenate was kept at RT/5 min, then centrifuged at 12000xg for 10 min at 4 oC. 300 µl of chloroform was applied to the fluid phase in a new tube, mixed vigorously for 15 s, before being incubated at RT/2 min. The tubes were centrifuged at 12,000 × g for 15 min at 4 oC. The RNA-containing aqueous layer was then transferred to a fresh tube, and 0.75 ml of isopropanol was added, thoroughly mixed by a gentle 10:15 inversion of the tubes. The tubes were centrifuged at 12,000 × g for 15 min at 4 oC, and the supernatant was then carefully removed. RNA washing was done with 1.5 ml of 75% ethanol, and the tubes were centrifuged at 7500 × g for 5 min at 4 oC. Following a 5-min air-drying period and a 56 °C/10–15-min incubation period with 50 µl or 100 µl DEPC-treated water to dissolve the RNA pellet, the samples were then stored at -20 °C until cDNA synthesis was completed.

Analyzing the samples' optical densities at 260 and 280 nm with a UV1100 spectrophotometer (Sciloge SP-UV1100, Marshall Scientific) was used to calculate both the RNA concentration and purity followed by gel electrophoresis. Tiny Genius BIOER thermal cycler was used to synthesize first strand cDNA from the extracted total RNA using the RevertAid™ First Strand cDNA Synthesis Kit (Thermo Scientific, USA). using 1 µl Oligo (dT)_18_ and 5 µg RNA sample, the mixture was incubated at 65^O^C for 5 min, after incubation 4 µl 5 × reaction buffer, 1 µl RiboLock RNase inhibitor (20U/ µl), 2 µl 10 mM dNTP mix, and 1 µl RevertAid m-MuLV RT (200U/ µl) were added to the mixture then the reaction was incubated at 42 °C / 60 min and the reaction was terminated at 70 oC /5 min. cDNA was diluted 1:4 with DNase /RNase free water.

### Gene expression analysis using Real-time PCR technique

For gene amplification, StepOne™ Applied Biosystem was utilized for real-time PCR. Each biological sample was analyzed in technical replicates of three for every target gene and reference gene. For each gene, appropriate specific forward and reverse primers were used. The GAPDH gene served as a reference for evaluating the expression levels of the target genes. The gene symbol, primer sequence, product size, accession number, and references of the primers are reviewed in Table 3.

**Table 3:** Gene-specific oligonucleotide primers used in real-time PCR

Reference	Accession NO	Product size	Primer sequence (5’ → 3’)	Primer name	Gene symbol
[61]	M16966	73 bp	CAGAAGCTCCAAGTCGCCTTT	F	LPL
			GACCCCCTGGTGAATGTGTG	R	
[56]	NM_174294.1	150 bp	TGCAATGATGAAGAGTTTTTTCCTAG	F	CSN3
			GATTGGGATATATTTGGCTATTTTGT	R	
[51]	GU324293.1		TCCACAAGCATCTTCGAGAAG	F	GLUT1
			AATAGCGACACGACAGTGAAG	R	
[56]	NM_001034034.1	197 bp	GTCTTCACTACCATGGAGAAGG	F	GAPDH
			TCATGGATGACCTTGGCCAG	R	

*F*  Forward, *R*  Reverse

The real-time PCR reaction was conducted in 8-strips of 0.1 ml qPCR with flat caps designed for real-time PCR systems. cDNA as a template, specific forward and reverse primers for each gene, and Maxima SYBER Green/ROX qPCR master mix were combined with a final volume of 20 µl for each reaction(10µl SYBER Green, 1.5µl forward primer (10pmol/µl), 1.5µl reverse primer (10pmol/µl), 3µl nuclease free water, and Finally 4µl cDNA sample then thoroughly mixed). The conditions required for the amplification of each gene are listed in Table 4. Using a melting curve, the product's specificity was examined. The 2^^−ΔΔCT^ method was utilized to evaluate the relative variances in gene expression based on the real-time quantitative PCR results [62].

**Table 4:** Real-time PCR amplification conditions

Gene symbol	Step	Temperature	Time	Cycles number
LPL	Denaturation	94 ^O^C	7 min	1
	Cycling program	94 ^O^C, 60 ^O^C	15 s, 30 s	35
CSN3	Denaturation	95 °C	5 min	1
	Cycling program	95 ^O^C, 62 ^O^C	30 s, 1 min	35
GLUT1	Denaturation	95 ^O^C	10 min	1
	Cycling program	95 ^O^C, 60 ^O^C, and 72 ^O^C	30 s, 30 s, 30 s	40

### Statistical analysis of data

Factorial experiment under general linear model’s assumptions was used for studying the effect of different dairy cattle breeds on milk components (Fat, Protein, and lactose) and gene expressions (LPL, GLUT1 and CSN3) under different stages of lactation, as mentioned in the following model:

$$y_{ijk}=\mu+B_i+L_j+{\left(BL\right)}_{ij}+\varepsilon_{ijk}$$

Where:

$y_{ijk}$; Dependent variables under the study (milk components, and gene expressions).

$\mu$; The overall mean.

$B_i$; Fixed breed effect (Three levels; Egyptian buffalo, red cow, and black cow).

$L_j$; Fixed lactation effect (Two levels; early stage, and peak stage).

${\left(BL\right)}_{ij}$; Interaction between breed and lactation stage.

$$\varepsilon_{ijk}$$; Random error.

The main effects and interactions were computed in two-way ANOVA procedures according to [63]. The pair wise comparison and mean separation were done by Duncan [64]. All statistical Analyses were carried out using IBM SPSS Statistics for Windows, version 26.0 (IBM Corp., Armonk, NY 1989–2019).

The use of single independent variable in regression analysis with more than o ne independent variable known as, multivariate regression model [65–68]. The multivariate linear regression model was developed to investigate the relationship between the gene expression of LPL, CSN3, and GLUT1 as independent variables and milk composition (fat%, protein %, and Lactose%) as follow:

$$y=\beta_0+B_iG_{LPL}+B_2G_{CSN3}+B_3G_{GLUT1}+\varepsilon$$

Where,

$y=$ observed values of the dependent variables (Fat%, Protein, and Lactose%).

$$\beta_0=$$ Constant

$\beta_{\left(1-3\right)}=$ unstandardised Regression coefficient for each predictor variable.

Predictor variables:

G_LPL_: LPL gene expression.

G_CSN3_: CSN3 gene expression.

G_GLUT1_: GLUT1 gene expression.

ε: Error.

Firstly, the regression assumptions normality, linearity, multicollinearity, missing and extreme values [66], were firstly tested using Statistical package for social survey (SPSS) ® 26.00 [69]**,** and was used for development of multivariate regression model.

Kolmogorov–Smirnov and Shapiro test were used to test normality assumption between dependent and independent variables. Mahalanobis distance versus chi-square used to check the extremes. The data also tested for linearity assumption between the outcome variables and explanatory ones using a simple scatter plot [70] and ANOVA. Finally, the multicollinearity among supposed predictors was tested using collinearity statistics (Tolerance, and variance inflation factor) [71].

## Data Availability

All data generated or analyzed during this study are included in this article.

## Abbreviations

NEFA: Non-esterified free fatty acids
ANOVA: Analysis of variance
Bp: Base pair
cDNA: Complementary deoxyribonucleic acid
DIM: Days in milk
FA: Fatty acid
GLUT: Glucose transporter
GLUT1: Glucose Transporters1
GAPDH: Glyceraldehyde-3-phosphate dehydrogenase
HF: Holstein-friesian
CSN3: Kappa casein
LCFA: Long chain fatty acids
LPL: Lipoprotein lipase
MECs: Mammary epithelial cells
mRNA: Messenger RNA
MFGs: Milk fat globules
PCR: Polymerase chain reaction
qPCR: Quantitative polymerase chain reaction
RNA: Ribonucleic acid
SPSS: Statistical package for the social sciences
TMR: Total mixed ration
TAG: Triglyceride
VLDL: Very low-density lipoprotein
RT: Room temperature

## References

[1] Boro P, Naha BC, Prakash C, Madkar A, Kumar N, Kumari A, Channa GP (2016). Genetic and non-genetic factors affecting milk composition in dairy cows. Int J Adv Biol Res.

[2] Kebede EJEJoAS (2018). Effect of cattle breed on milk composition in the same management conditions. J Ethiopian J Agric Sci.

[3] Kailasapathy K (2015). Chemical composition, physical, and functional properties of milk and milk ingredients. Dairy processing and quality assurance.

[4] Kern PA, Mandic A, Eckel RHJ (1987). Regulation of lipoprotein lipase by glucose in primary cultures of isolated human adipocytes: relevance to hypertriglyceridemia of diabetes. Diabetes.

[5] Jensen DR, Gavigan S, Sawicki V, Witsell DL, Eckel RH, Neville MCJBJ (1994). Regulation of lipoprotein lipase activity and mRNA in the mammary gland of the lactating mouse. Biochem J.

[6] Jensen D, Bessesen D, Etienne J, Eckel R, Neville MJ (1991). Distribution and source of lipoprotein lipase in mouse mammary gland. J Lipid Res.

[7] Moioli B, D’Andrea M, Pilla FJ (2007). Candidate genes affecting sheep and goat milk quality. Small Rumin Res.

[8] Martin M, Eckel Robert H, Goldberg Ira JJJ (2002). Lipoprotein lipase: genetics, lipid uptake, and regulation. Lipid Res.

[9] Sekar R, Selvasekaran P, Kar A, Varalwar T, Godli C, Chidambaram R (2020). Lactose-free food products for lactose intolerant children. Food science, technology nutrition for babies children.

[10] Costa A, Lopez-Villalobos N, Sneddon N, Shalloo L, Franzoi M, De Marchi M, Penasa M (2019). Invited review: Milk lactose—Current status and future challenges in dairy cattle. J Dairy Sci.

[11] Lacroix M, Bos C, Léonil J, Airinei G, Luengo C, Daré S, Benamouzig R, Fouillet H, Fauquant J, Tomé D (2006). Compared with casein or total milk protein, digestion of milk soluble proteins is too rapid to sustain the anabolic postprandial amino acid requirement. Am J Clin Nutr.

[12] Singh H (2011). Aspects of milk-protein-stabilised emulsions. Food Hydrocolloids.

[13] Banach JC, Clark S, Lamsal BP (2016). Microstructural changes in high-protein nutrition bars formulated with extruded or toasted milk protein concentrate. J Food Sci.

[14] Foroutan A, Guo AC, Vazquez-Fresno R, Lipfert M, Zhang L, Zheng J, Badran H, Budinski Z, Mandal R, Ametaj BN (2019). Chemical composition of commercial cow’s milk. J Agric Food Chem.

[15] Fox P, Uniacke-Lowe T, McSweeney P, O'Mahony J, Fox P, Uniacke-Lowe T, McSweeney P, O’Mahony J (2015). Milk proteins. Dairy Chemistry Biochemistry.

[16] Sarkar U, Gupta A, Sarkar V, Mohanty T, Raina VS, Prasad S (2006). Factors affecting test day milk yield and milk composition in dairy animals. J Dairying Foods Home Sci.

[17] Kayastha R, Zaman G, Goswami R (2008). Factors affecting the milk constituents of native cattle of Assam. Indian J Anim Res.

[18] Osorio JS, Lohakare J, Bionaz M (2016). Biosynthesis of milk fat, protein, and lactose: roles of transcriptional and posttranscriptional regulation. Physiol Genomics.

[19] Bionaz M, Loor JJ (2008). Gene networks driving bovine milk fat synthesis during the lactation cycle. BMC Genomics.

[20] Sandri E, Harvatine K, Oliveira D (2020). Trans-10, cis-12 conjugated linoleic acid reduces milk fat content and lipogenic gene expression in the mammary gland of sows without altering litter performance. Br J Nutr.

[21] Gutierrez-Reinoso M, Aponte P, Garcia-Herreros M (2021). Genomic analysis, progress and future perspectives in dairy cattle selection: a review. Animals.

[22] Zhao W-S, Hu S-L, Yu K, Wang H, Wang W, Loor J, Luo JJ (2014). Lipoprotein lipase, tissue expression and effects on genes related to fatty acid synthesis in goat mammary epithelial cells. Int J Mol Sci.

[23] Kucerova J, Matejicek A, Jandurová O, Sorensen P, Nemcova E, Stipkova M, Kott T, Bouska J, Frelich JJ (2006). Milk protein genes CSN1S1, CSN2, CSN3, LGB and their relation to genetic values of milk production parameters in Czech Fleckvieh. Czeh J Anim Sci.

[24] Alexander LJ, Stewart AF, Mackinlay AG, Kapelinskaya TV, Tkach TM, Gorodetsky SIJ (1988). Isolation and characterization of the bovine k-casein gene. Eur J Biochem.

[25] Sørensen J, Palmer DS, Qvist KB, Schiøtt BJ (2011). Initial stage of cheese production: a molecular modeling study of bovine and camel chymosin complexed with peptides from the chymosin-sensitive region of κ-casein. J Agric Food Chem.

[26] Looney MA. Characterization of changes in composition and physicochemical properties of casein micelles from raw milk to buttermilk. California Polytechnic State University; 2014. 10.15368/theses.2014.13. Available at: https://digitalcommons.calpoly.edu/theses/1162.

[27] Lin Y, Sun X, Hou X, Qu B, Gao X, Li QJ (2016). Effects of glucose on lactose synthesis in mammary epithelial cells from dairy cow. BMC Vet Res.

[28] Zhao F-Q, Keating AJ (2007). Expression and regulation of glucose transporters in the bovine mammary gland. J Dairy Sci.

[29] Wang YH, Bower N, Reverter A, Tan S, De Jager N, Wang R, McWilliam S, Cafe L, Greenwood P, Lehnert SJ (2009). Gene expression patterns during intramuscular fat development in cattle. J Anim Sci.

[30] Yadav P, Singh DD, Mukesh M, Kataria R, Yadav A, Mohanty A, Mishra BJ (2014). Expression profiling of glucose transporter 1 (GLUT1) and apoptotic genes (BAX and BCL2) in milk enriched mammary epithelial cells (MEC) in riverine buffalo during lactation. Anim Biotechnol.

[31] Dulbecco R, Henahan M, Armstrong B (1982). Cell types and morphogenesis in the mammary gland. Proc Natl Acad Sci.

[32] Maningat PD, Sen P, Rijnkels M, Sunehag AL, Hadsell DL, Bray M, Haymond MW (2009). Gene expression in the human mammary epithelium during lactation: the milk fat globule transcriptome. Physiol Genomics.

[33] Van Keymeulen A, Rocha AS, Ousset M, Beck B, Bouvencourt G, Rock J, Sharma N, Dekoninck S, Blanpain C (2011). Distinct stem cells contribute to mammary gland development and maintenance. Nature.

[34] Lemay DG, Neville MC, Rudolph MC, Pollard KS, German JB (2007). Gene regulatory networks in lactation: identification of global principles using bioinformatics. BMC Syst Biol.

[35] Patton S, Huston GE (1988). Incidence and characteristics of cell pieces on human milk fat globules. Biochim Biophys Acta -Gen Subj.

[36] McManaman JL, Palmer CA, Anderson S, Schwertfeger K, Neville MC (2004). Regulation of milk lipid formation and secretion in the mouse mammary gland. Protecting infants through human milk: advancing the scientific evidence.

[37] Boutinaud M, Herve L, Lollivier V (2015). Mammary epithelial cells isolated from milk are a valuable, non-invasive source of mammary transcripts. Front Genet.

[38] Maningat PD, Sen P, Sunehag AL, Hadsell DL, Haymond MW (2007). Regulation of gene expression in human mammary epithelium: effect of breast pumping. J Endocrinol.

[39] Brenaut P, Bangera R, Bevilacqua C, Rebours E, Cebo C, Martin P (2012). Validation of RNA isolated from milk fat globules to profile mammary epithelial cell expression during lactation and transcriptional response to a bacterial infection. J Dairy Sci.

[40] Cánovas A, Rincón G, Bevilacqua C, Islas-Trejo A, Brenaut P, Hovey RC, Boutinaud M, Morgenthaler C, VanKlompenberg MK, Martin P (2014). Comparison of five different RNA sources to examine the lactating bovine mammary gland transcriptome using RNA-Sequencing. Sci Rep.

[41] Sharma A, Shandilya UK, Sodhi M, Jatav P, Mohanty A, Jain P, Verma P, Kataria R, Kumari P, Mukesh M (2019). Milk-derived mammary epithelial cells as non-invasive source to define stage-specific abundance of milk protein and fat synthesis transcripts in native Sahiwal cows and Murrah buffaloes. 3 Biotech.

[42] Garau V, Manis C, Scano P, Caboni P (2021). Compositional characteristics of mediterranean buffalo milk and whey. Dairy.

[43] Janmeda M, Kharadi V, Pandya G, Brahmkshtri B, Ramani U, Tyagi K (2017). Relative gene expression of fatty acid synthesis genes at 60 days postpartum in bovine mammary epithelial cells of Surti and Jafarabadi buffaloes. Vet World.

[44] Wickramasinghe S, Rincon G, Islas-Trejo A, Medrano JF (2012). Transcriptional profiling of bovine milk using RNA sequencing. BMC Genomics.

[45] Arora R, Sharma A, Sharma U, Girdhar Y, Kaur M, Kapoor P, Ahlawat S, Vijh RK (2019). Buffalo milk transcriptome: a comparative analysis of early, mid and late lactation. Sci Rep.

[46] Ahlawat S, Arora R, Sharma U, Sharma A, Girdhar Y, Sharma R, Kumar A, Vijh RK (2021). Comparative gene expression profiling of milk somatic cells of Sahiwal cattle and Murrah buffaloes. Gene..

[47] Contreras GA, Strieder-Barboza C, Raphael W (2017). Adipose tissue lipolysis and remodeling during the transition period of dairy cows. J Anim Sci Biotechnol.

[48] Zhao F-Q, Keating AF (2007). Functional properties and genomics of glucose transporters. Curr Genomics.

[49] Dänicke S, Meyer U, Kersten S, Frahm J (2018). Animal models to study the impact of nutrition on the immune system of the transition cow. Res Vet Sci.

[50] Mann S, Jatav P, Sodhi M, Shandilya U, Sharma A, Mukesh M (2014). An overview of solute carrier family (facilitated glucose transporter) genes and their role in bovine mammary gland functioning. Int J Anim Biotech.

[51] Pradeep J, Monika S, Ankita S, Umesh K, Amit K, Ashok M, Mishra B, Sandeep M, Kataria R, Kaushik J (2014). Expression analysis of solute carrier (SLC2A) genes in milk derived mammary epithelial cells during different stages of lactation in sahiwal (Bos indicus) cows. Adv Dairy Res.

[52] Mepham TB (1983). Biochemistry of lactation.

[53] Sadovnikova A, Garcia SC, Hovey RC (2021). A comparative review of the extrinsic and intrinsic factors regulating lactose synthesis. J Mammary Gland Biol Neoplasia.

[54] Cant JP, Trout DR, Qiao F, Purdie NG (2002). Milk synthetic response of the bovine mammary gland to an increase in the local concentration of arterial glucose. J Dairy Sci.

[55] Kronfeld DS (1982). Major Metabolic Determinants of Milk Volume, Mammary Efficiency, and Spontaneous Ketosis in Dairy Cows1. J Dairy Sci.

[56] Sigl T, Meyer HHD, Wiedemann S (2012). Gene expression of six major milk proteins in primary bovine mammary epithelial cells isolated from milk during the first twenty weeks of lactation. Czeh J Anim Sci.

[57] Bhat SA, Ahmad SM, Ibeagha-Awemu EM, Bhat BA, Dar MA, Mumtaz PT, Shah RA, Ganai NA (2019). Comparative transcriptome analysis of mammary epithelial cells at different stages of lactation reveals wide differences in gene expression and pathways regulating milk synthesis between Jersey and Kashmiri cattle. PLoS ONE..

[58] Gáspárdy A, Schwartz Z, Zöldág L, Veresegyházy T, Fekete SJ (2004). Changes in daily energy amounts of main milk components (lactose, protein and fat) during the lactation of high-yielding dairy cows. Acta Vet Hung.

[59] Wiame I, Remy S, Swennen R, Sági L (2000). Irreversible heat inactivation of DNase I without RNA degradation. Biotechniques.

[60] Sharma A, Girdhar Y, Kaur M, Ahlawat S, Vijh RK, Arora RJ (2018). Evaluation of milk fat as an alternative source of RNA from buffalo milk. Buffalo Bull.

[61] Miller N, Delbecchi L, Petitclerc D, Wagner G, Talbot B, Lacasse PJ (2006). Effect of stage of lactation and parity on mammary gland cell renewal. J Dairy Sci.

[62] Livak KJ, Schmittgen TD (2001). Analysis of relative gene expression data using real-time quantitative PCR and the 2− ΔΔCT method. Methods..

[63] Snedecor GW, Cochran WG (1989). Statistical methods.

[64] Duncan DB (1955). Multiple range and multiple F tests. Biometrics..

[65] Acet H (2016). The relationship between capital formation and economic growth of Niger (1980–2014). In: GAI International Academic Conferences Proceedings.

[66] Büyüköztürk Ş (2018). Sosyal bilimler için veri analizi el kitabı. Pegem Atıf İndeksi.

[67] Köksal BA: İstatistik analiz metodları: Boğaziçi Üniversitesi Yayınları; 1977.

[68] Tabachnick BG, Fidell LS (1996). Using multivariate statistics.

[69] Misra A, Roy S, Singh S, Rathi R, Harish G (2019). Morphological diversity of buckwheat (Fagopyrum spp.) landraces from Northeast India. Indian J Plant Genet Res..

[70] Utts JM (2014). Seeing through statistics: cengage learning.

[71] Abdelrahman HA, Kamel ER, Hassan FA (2020). Application of linear discriminant model to evaluate the association between milk production, reproductive performance, and calving season in dairy cattle. Benha Vet Med J.

